# Fuling Sini decoction for patients with chronic heart failure: A protocol for a systematic review and meta-analysis

**DOI:** 10.1097/MD.0000000000013692

**Published:** 2018-12-21

**Authors:** Lijie Huang, Hairong Cai, Jieqin Zhuang, Yanhong Chen, Zilin Jin, Haobo Zhang, Huanjia Gao

**Affiliations:** a The Basic Medicine College of Guangzhou University of Chinese Medicine; b The Second Clinical Medical College of Guangzhou University of Chinese Medicine, Guangzhou, Guangdong Province, China.

**Keywords:** chronic heart failure, Fuling Sini decoction, protocol, systematic review

## Abstract

**Background:**

Chronic heart failure (CHF) is one of the most important cardiovascular diseases worldly, with high morbidity and mortality. Fuling Sini decoction (FSD) has been used in the management of CHF widely in China, while its effective evidence is not clear. A systematic review and meta-analysis to evaluate FSD for CHF is lacking. Hence, we propose a protocol for systematic evaluation of its efficacy and safety for CHF.

**Methods:**

We will search the following electronic databases from inception to October 2018, including EMBASE, Cochrane Center Registration Controlled trials (Cochrane Library), PubMed, Medline, WHO International Clinical Trials Registry Platform, China Biomedical Literature Database (CBM), China National Knowledge Infrastructure (CNKI), Chinese Scientific Journal Database (VIP), and Wan-fang database. Only randomized controlled trials (RCTs) of FSD alone or combined with other management for CHF will be included. Two independent reviewers will screen the literature and extract data according to the Cochrane Handbook method. The assessment of bias risk, data synthesis, subgroup analysis, and meta-analyses and finally meta-analysis will be performed by using RevMan V.5.3.5 software.

**Results:**

This systematic review will provide high-quality evidence and may be the first to evaluate efficacy and safety of FSD in the treatment of CHF.

**Conclusion:**

This systematic review will provide evidence for judging whether FSD is an effective management for CHF or not.

**PROSPERO registration number:**

CRD 42018110210.

## Abstract 2

Supplemental Digital Content is available in the text

## 1 Introduction

Chronic heart failure (CHF) is a complex clinical syndrome in which ventricular filling or impaired ejection capacity is impaired by any abnormal cardiac structure or function, with main manifestations of dyspnea, fatigue, and fluid shift (pulmonary congestion and peripheral edema).^[1]^ CHF is a serious and terminal stage of various heart diseases, and one of the most important cardiovascular diseases, with high morbidity and mortality. The total number of patients with CHF reached 22.5 million worldly with its incidence increasing year by year. In the United States, the prevalence of CHF is 3.8‰ in the male population, and 2.9‰ in female.^[2]^ There were about 5 million people accounting for 1.5% to 2.0%, and still continue to increase at a rate of 550,000/year.^[3]^ CHF was the leading cause of hospitalization and its annual cost was as high as 33 billion US dollars, causing a great social burden.^[4]^ In Europe, there are an estimated 1 million patients with CHF, accounting for 0.4% to 2% of the total population.^[5]^ In China, there were 4 million patients with CHF, and the prevalence rate is 0.9%, the prevalence of women (1.0%) is higher than that of men (0.7%), north (1.4%) higher than South (0.5%), cities (1.1%) higher than rural areas (0.8%). The prevalence is significantly increased with the increase of age, and the prevalence of CHF in people (65–74 age) is about 1.3%.^[6]^ The mortality rate of hospitalized patients was 5.3%,^[7]^ and the 5-year mortality rate was about 50%, similar to malignant tumors.^[2,8]^ Once suffering from CHF, the patient will experience reduced activity, which severely affects their quality of life. CHF has become one of the focuses of cardiovascular disease in the 21st century.

Traditional Chinese medicine (TCM) is an important part of complementary and alternative medicine (CAM), which has been widely accepted and applied in clinical practice in China.^[9]^ With the advances of modern medicine in the concept and means of treatment for CHF, prognosis of patients with CHF has been significantly improved; however, TCM has been widely used in the treatment of CHF because of its advantages in stabilizing the disease, improving heart function and the quality of life.^[10,11]^ Fuling Sini decoction (FSD) is comprised of the following 5 kinds of Chinese medicines: Fuling (Poria Cocos (schw) Wolf), Renshen (Ginseng radix et rhizoma), Gancao (Glycyrrhizae radix et rhizoma), Fuzi (Aconitum carmichaeli Debx), Ganjiang (Rhizoma Zingiberis). Clinical studies have shown that FSD could improve quality of life, reduce edema, improve patients’ New York Heart Association (NYHA) heart functional classification, improve left ventricular ejection fraction (LVEF), and reduce aminoterminal pro-brain natriuretic peptide (NT-proBNP) levels.^[12,13]^ However, there is currently no systematic review regarding its efficacy and safety in the treatment of CHF. Hence, we propose this protocol of systematic review and meta-analysis to assess the efficacy and safety of FSD for CHF, and thus provides a reference for the use of FSD in clinical practice.

## 2 Methods

### 2.1 Inclusion criteria for study selection

#### 2.1.1 Types of studies

Only randomized controlled trials (RCTs) of FSD on CHF will be included, without restrictions on blinding, language, and publication time. Letters, comments, case reports, and case series will be excluded.

#### 2.1.2 Types of patients

Patients (18 years of age and older) with NYHA heart function classification II ∼ IV grade and diagnosed CHF will be included. The diagnostic criteria for CHF will be developed according to the 22013 ACCF/AHA Guideline for the Management of Heart Failure: A Report of the American.^[14]^ The primary disorder of CHF includes coronary heart disease, rheumatic heart disease, dilated cardiomyopathy, and hypertensive heart disease. There is no limitation on age, gender, race, nationality, and comorbidity. Patients with acute myocardial infarction (AMI) and unstable angina (UA) in the past 3 months, history of cardiac surgery, stroke, or TIA in the past 6 months, severe liver and kidney dysfunction, cardiogenic shock, end-stage cancer, and severe ventricular arrhythmia will be excluded.

#### 2.1.3 Types of interventions

Patients in the experimental group have been treated with conventional management for CHF [including diuretics, angiotensin-converting enzyme inhibitors (ACEIs) or angiotensin II receptor blockers (ARBs), the β-blockers, digitalis preparations, aldosterone receptor antagonists, nitrates, etc] combined with FSD, and the control group treated with conventional management or combined with placebo. The administration time of each group of patients is not less than 6 months.

#### 2.1.4 Types of outcome measures

##### 2.1.4.1 Primary outcomes

Primary outcomes included mortality or hospital readmission rate at the end of the follow-up period (at least 1 year).

##### 2.1.4.2 Secondary outcomes

Secondary outcomes included clinical efficacy: markedly effective: NYHA heart function classification improve grade 2 or higher, symptoms and signs relief completely after treatment; effective: NYHA heart function classification improve grade 1, symptoms and signs relief partially; invalid: NYHA heart function classification improve less than grade 1, no relief of symptoms and signs or even worse; NYHA heart function classification; LVEF; left ventricular mass index (LVMI); NT-proBNP; blood pressure (BP); weight; 6-minute walking test; heart rate; adverse events; and all-cause death.

#### 2.1.5 Ethics approval and patient consent

This study is based on published data study as such ethics approval is not a requirement. This study is a systematic review, it will not include patient, and patient consent is not a requirement.

### 2.2 Search methods for the identification of studies

A search will be performed in the following electronic databases from inception to October 2018, including EMBASE, Cochrane Center Registration Controlled trials (Cochrane Library), PubMed, Medline, WHO International Clinical Trials Registry Platform, China Biomedical Literature Database (CBM), China National Knowledge Infrastructure (CNKI), Chinese Scientific Journal Database (VIP), and Wan-fang database by a combination of subject words and free words. The search terms included congestive heart failure, heart or cardiac failure, RCT, and random. The strategy for searching the PubMed will be shown as an example in Appendix A (Supplemental Appendix A), and modified by using other databases.

#### 2.2.1 Searching other resources

Meanwhile, we will search relevant journals and follow-up the relevant literature in the reference to avoid missing any relevant studies, and use the search engines such as Google Scholar to find relevant documents on the Internet manually. In the meantime, we will search Clinical Trials. Gov for completed but unpublished research and track the findings.

### 2.3 Data collection and analysis

#### 2.3.1 Selection of studies

**Figure 1:**
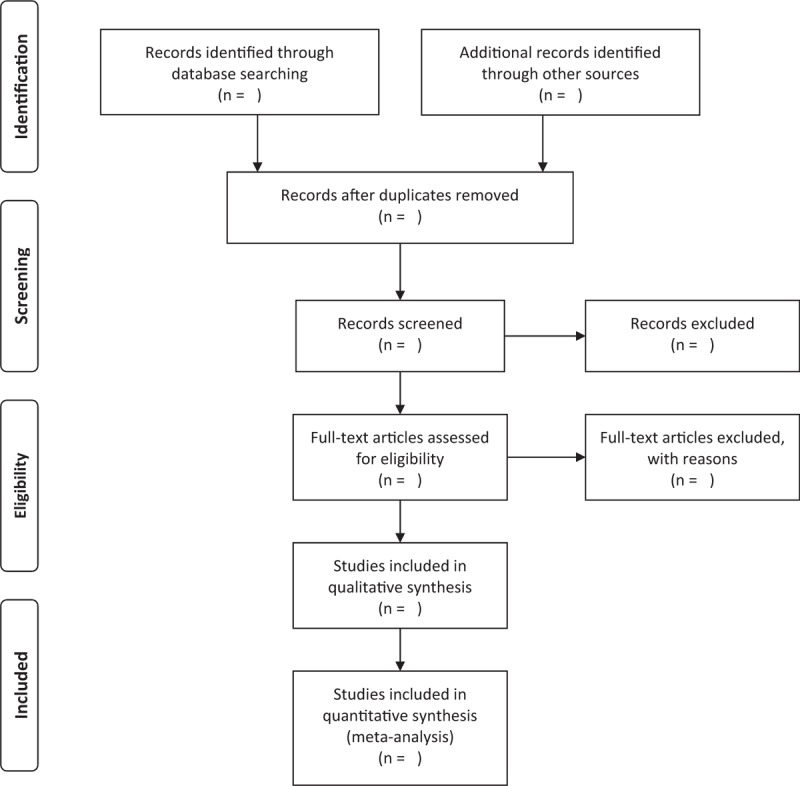
Preferred reporting items for systematic review and meta-analysis (PRISMA) flow chart.

Two review authors will screen the literature according to the predetermined inclusion criteria and exclusion criteria independently. In the initial process of selection, the authors will scan the titles and abstracts of literature and collect all possible relevant and certain related researches. In the second stage of selection, the 2 authors will read the full text of articles and confirm the eligibility for our review. The results will be double checked for accuracy. The reasons for inclusion or exclusion of each article will be recorded. Where there is any disagreement, it could be resolved by discussion or consulting with a third review author. The process of studies selection and meta-analysis is presented in a in an adapted PRISMA flow diagram (Fig. 1).

#### 2.3.2 Data extraction and management

Two independent authors will extract information by using standardized extraction form, including general information (age, gender, sample size, etc), intervention measures (dosage types of the experimental group and the control group, administration dose, frequency of administration, course of treatment, etc), and outcome indicators. Then, they will check the results for accuracy doubly. Any disagreement could be resolved through discussion or consulting with a third author. If information is missing, the authors will contact the corresponding author and first author by phone or email to obtain the necessary data.

#### 2.3.3 Assessment of risk of bias in included studies

The evaluation of quality will be conducted according to the Risk of bias tool recommended by Cochrane Handbook 5.3.1 (The Cochrane Collaboration, Oxford, UK). The evaluation content includes generation of random sequences, randomization concealment, the implementation of blinded subjects and researchers, the implementation of blind methods for outcome evaluators, the integrity of outcome data, selective reporting, and other biases. Each item should be judged into 3 levels of “low risk bias,” “high risk bias,” and “unclear” in accordance with the quality classification criteria. For each item, if it is satisfied, it means “low risk bias,” and if it is not, it means “high risk bias.” When there is not enough information in the literature to make a clear judgment on the corresponding item, it means “unclear.” The assessment of risk bias will be assessed by 2 authors independently. If there is a disagreement, it will be resolved through discussion or by a third author.

#### 2.3.4 Measures of treatment effect

Dichotomous data will be presented as relative data (RR) with 95% confidence intervals (95% CIs), and continuous data will be presented as weighted mean difference (WMD) with 95% CI. When the units of continuous data are different, standardized mean difference (SMD) with 95% CI will be presented.

#### 2.3.5 Dealing with missing data

If the information of the article is missing, we will contact the author for further information. If the necessary information is not obtained, we will use the available data for data synthesis. At the same time, we will also discuss the possible consequences of missing data.

#### 2.3.6 Assessment of heterogeneity

The heterogeneity will be analyzed by using Chi-square test and the *I*^2^ statistic. *I*^2^ < 25% will be considered that there is no statistical heterogeneity or heterogeneity is small; *I*^2^ > 25% and <50% will be taken as moderate heterogeneity, while *I*^2^ > 50% will be taken as high heterogeneity. In cases of high heterogeneity, a subgroup analysis will be performed to investigate the underlying causes from clinical or methodological heterogeneity. A descriptive analysis will be performed in case of clinical heterogeneity persists.

#### 2.3.7 Assessment of reporting bias

If more than 10 articles are included, we will analyze the impact of publication bias based on the funnel plot of Revman Manager software (version 5.3; The Cochrane Collaboration, Oxford, UK).

#### 2.3.8 Data synthesis

The meta-analysis will be performed by using RevMan5.3 software provided by the Cochrane Collaboration. A fixed effect model will be used if there is no statistical heterogeneity or heterogeneity is low (*I*^2^ ≤ 50%). If there is significant heterogeneity (*I*^2^ > 50%), subgroup analysis and sensitivity analysis will be performed to find the causes from clinical heterogeneity or statistical heterogeneity. A random effects model will be used for meta-analysis in case of statistical but no clinical heterogeneity. Descriptive analysis will be performed if it is not available to conduct a meta-analysis. a = 0.05 will be deemed statistically significant.

#### 2.3.9 Subgroup analysis

Subgroup analysis is to explore the source of heterogeneity. When more than 10 studies are included, subgroup analyses can be performed according to interventions, participants, age, gender, duration of disease, and dose.

#### 2.3.10 Sensitivity analysis

Sensitivity analysis will be carried out to examine the robustness of the pooled results in case of sufficient data, by determining the effects of excluding studies with high risks of bias, studies with missing data, and outliers.

#### 2.3.11 Grading the quality of evidence

It is recommended to use the Grading of Recommendations Assessment, Development and Evaluation (GRADE)^[15,16]^ to analyze the quality level of evidence. The results will be divided into 4 levels: high, medium, low or very low, and the recommended level will be combined with the topic.

## 3 Discussion

CHF is one of the leading causes of death in humans. Diuretics, ACEIs, beta blockers, and ARB are considered to be standard management for CHF, which could reduce cardiac load and delay myocardial remodeling to maximize cardiac function.^[17]^ The goal of treating CHF is delaying the progression of the disease, alleviating clinical symptoms, improving long-term prognosis and quality of life, and reducing mortality. FSD has been widely used in treatment of patients with CHF in clinical practice. A large number of studies have shown that FSD could improve the quality of life and NYHA heart function classification, and reduce BNP levels in patients with CHF. However, a high quality of systematic review regarding its efficacy and safety in the treatment of CHF is lacking. Therefore, we designed this systematic review and meta-analysis for evaluating efficacy and safety of FSD for CHF, hoping this study will provide more convincing evidence to demonstrate the advantages of FSD for CHF and provide reference for clinical practice. However, there may be some potential shortcomings in this study. First, different doses of herbs, patient age, and severity of CHF may present a heterogeneity risk. Finally, small samples of trails may lead to high risks of bias.

## Author contributions

**Conceptualization:** Lijie Huang, Hairong Cai.

**Data curation:** Jieqin Zhuang.

**Investigation:** Yanhong Chen, Zilin Jin.

**Software:** Huanjia Gao.

**Supervision:** Lijie Huang, Hairong Cai.

**Validation:** Haobo Zhang.

**Writing – original draft:** Lijie Huang, Hairong Cai.

## Supplementary Material

Supplemental Digital Content
