# Supplementary material for: Fuling Sini decoction for patients with chronic heart failure: A protocol for a systematic review and meta-analysis
Source: Medicine (Baltimore). 2018 Dec 21;97(51):e13692. doi: 10.1097/MD.0000000000013692 (PMC6320152; doi:10.1097/MD.0000000000013692)
Supplement: Supplemental Digital Content [file medi-97-e13692-s001.docx]

**Appendix A.**

***Search strategy used in PubMed database***

#1 congestive heart failure OR Cardiac Failure OR Heart Decompensation OR Decompensation, Heart OR Heart Failure, Right-Sided OR Heart Failure, Right Sided OR Right-Sided Heart Failure OR Right Sided Heart Failure OR Myocardial Failure OR Heart Failure, Congestive OR Heart Failure, Left-Sided OR Heart Failure, Left Sided OR Left-Sided Heart Failure OR Left Sided Heart Failure

#2 fuling sini decoction OR fu ling si ni decoction OR fuling sini tang OR fuling sini yin

#3 Randomized controlled trial OR clinical study OR Clin-ical Trial OR Controlled study OR Controlled Trial OR Random*Control* study OR random* Control* Trial

#1 AND #2 AND #3
